# Effects of Mindful Practices on Terror of Mortality: A Randomized Controlled Trial

**DOI:** 10.1007/s12671-022-01967-8

**Published:** 2022-09-05

**Authors:** Bhikkhu Anālayo, Oleg N. Medvedev, Nirbhay N. Singh, Marie R. Dhaussy

**Affiliations:** 1 Numata Center for Buddhist Studies, University of Hamburg, Alsterterrasse 1, 20354 Hamburg, Germany; 2 University of Waikato, Te Whare Wananga o Waikato, Gate 1, Knighton Road, Hamilton, 3240 New Zealand; 3 Department of Psychiatry and Health Behavior, Medical College of Georgia, Augusta University, 997 St. Sebastian Way, Augusta, GA 30912 USA; 4 Medical School Hamburg, Am Kaiserkai 1, 20457 Hamburg, Germany

**Keywords:** Anxiety, Deathless, Fear of death, Mindfulness and fear, Mortality salience, Recollection of death, Self-compassion, Terror management theory

## Abstract

**Objectives:**

Early Buddhist thought clearly recognizes the need for learning how to face one’s own mortality, for which purpose mindfulness practice has a central role to play. Fear of death has also been studied in cognitive psychology, leading to what is known as the terror management theory. Actual research evidence in psychology has already shown that mindfulness practice may reduce fear and anxiety in general. However, there is a lack of research examining the specific effects of brief mindfulness practices on the fear of death and dying. In this study we tested the hypothesis that brief mindfulness practices used daily over a period of 6 weeks will result in a reduction of the fear of death and dying when compared to brief contemplative practices used as an active control condition.

**Methods:**

Participants (*n* = 89) were randomly assigned to the mindfulness (*n* = 44) and the contemplation (*n* = 45) conditions and completed validated scales measuring four distinct fears related to either the process of dying or the final event of death (dying of oneself, death of oneself, dying of others, and death of others), mindfulness, and self-compassion at baseline, post-intervention (at 6 weeks) and follow up (1‒3 weeks after the end of the 6-week intervention). ANOVA was used to investigate the effects of both interventions on outcome variables over time and between groups.

**Results:**

Both mindfulness and contemplative practices were equally effective in reducing fear related to dying of oneself and death of others while increasing fear of dying of others, mindfulness, and self-compassion. No significant intervention effects were found for fear related to death of oneself only.

**Conclusions:**

These results suggest that fears related to dying of oneself and death of others can be reduced using both mindfulness and contemplative practices that may simultaneously increase mindfulness and self-compassion.

**Supplementary Information:**

The online version contains supplementary material available at 10.1007/s12671-022-01967-8.

A gravestone in York, England, reportedly carries the following inscription (Brooks, 2005):

Remember, friend, when passing by,

As you are now, so once was I.

As I am now, [so] you will be.

Prepare for death and follow me.

The inscription reflects a perennial challenge of human life, which is none other than learning to face one’s own mortality, to allow death to become part of one’s life, rather than being ignored until the last moment. Much of the teachings given by different religious traditions revolve around attempts to provide some answer to this challenge. The above inscription inspired someone else to scribble a poetic reply on the gravestone, which succinctly captures the basic uncertainty that stands in the background of the pervasive tendency to ignore death:

To follow you I’m not content,

Until I know which way you went.

The challenge posed by death features among the central dimensions of the human predicament that, according to the *Ariyapariyesanā-sutta* and its Chinese parallel (MN 26 and MĀ 204), motivated the Buddha to go forth, together with the related problems of being subject to old age and disease. Another discourse extant similarly in Pāli and Chinese reports in detail the reflection that the Buddha-to-be developed at a time in his youth before going forth to live the life of a wandering mendicant. The reflection begins by describing how ignorant persons tend to overlook these features of the human predicament. Being faced with others who are sick, become old, or pass away, such persons just turn away in disgust, forgetting the fact that they are themselves subject to the same fate (AN 3.38 and MĀ 117; see also Anālayo 2017). In contrast to such ignorant avoidance, the Buddha-to-be clearly realized that he was not exempt from these predicaments.

With awakening successfully achieved, according to the *Ariyapariyesanā-sutta* and the same Chinese parallel the Buddha announced his discovery to those who were to become his first disciples by proclaiming that he had realized the “deathless” (*amata*/無死). The early Buddhist conception of the deathless involves a perspective that significantly differs from the employment of the same term among some contemporaries of the Buddha (Kumoi 1969; Vetter 1995). Instead of referring to a form of immortality, in its Buddhist usage the full realization of the deathless represents total freedom from any fear of death, to be accomplished while still alive. In fact, despite having realized the deathless, the Buddha still had to pass away. His body was not beyond being affected by old age, disease, and death. Yet, his mind was completely beyond being affected by the impact of old age, disease, and death on his body.

A Pāli discourse with a range of parallels report that the Buddha intentionally let go of his life force, even though in principle he would have been able to live much longer (Waldschmidt 1944, p. 101). At this point in his life, he had successfully achieved his mission of teaching others and establishing them in the realization of the deathless, thereby enabling them to be self-reliant. Since apparently nobody had made a special request for him to continue staying alive, the time had come for him to let go of an old and frail body.

The textual sources depict the Buddha’s final moments in detail. After giving a last teaching to his disciples, the Buddha is on record for proceeding through a whole range of meditative attainments. These cover the four absorptions, the four immaterial spheres, and the cessation of perception and feeling tone, attained in forward and again in reverse order (Anālayo 2014). The ability to gain each of these successively features as a mark of highest meditative accomplishment. The central message underlying this description is that the Buddha faced his own death with complete composure and in full possession of his meditative mastery of the mind.

In view of the precedent set by the Buddha’s example, it is perhaps no surprise to find that mortality and its repercussions are an important theme in early Buddhist meditation. Such a form of practice can be seen as a particularly powerful implementation of the general theme of directing mindfulness to a recognition of impermanence (Fernández-Campos et al. 2021). Out of the central dimensions of the human predicament that reportedly motivated the Buddha to go forth, death is the one topic that features as a theme for meditative recollection (Anālayo 2022a). The cultivation of recollection as such is a mindfulness practice (Anālayo 2020), which in the present case involves recollecting something bound to happen in the future, namely one’s own passing away. This is of interest as, contrary to the impression perhaps evoked by the English term “recollection” (Pāli *anussati*; Sanskrit *anusmṛti*; Chinese 憶念; Tibetan *rjes su dran pa*), the actual practice is not confined to calling up memories from the past.

A chief challenge in executing this type of practice is the natural human tendency of assuming that one’s own death will only occur at some rather distant time in the future. A Pāli discourse and its Chinese parallel report the Buddha directly tackling this tendency among some of his disciples. According to the introductory narration, the Buddha had inquired into how these disciples were practicing mindfulness of death (AN 6.19 / AN 8.73 and EĀ 40.8). On finding that their practice involved imagining that death was going to come at a more or less distant time in the future, the Buddha concluded that they were being negligent. According to his instruction, mindfulness of death should be practiced with the thought that it could happen with the next breath. In other words, there needs to be the clear acknowledgement that death can in principle happen right here and now.

The advice given in this way features among a range of instructions related not only to one’s own death but also to the death of others (Anālayo 2016), even to the extent of offering detailed suggestions for palliative care (SN 55.54 and SĀ 1122). The repercussions of mortality are also an integral part of the instructions on the systematic development of mindfulness in the *Satipaṭṭhāna-sutta* and its parallels (Anālayo 2013). The relevant contemplation depicts a human corpse in various stages of decay, a vision of which should lead the meditator to the basic insight that the Buddha-to-be realized in his youth, namely the fact of being oneself subject to the same fate. The actual instructions appear to some extent to involve an element of visualization (Anālayo 2022b), perhaps reflecting the importance of making death as palpable as possible to ensure that its inevitable impact sinks into the mind of the meditator and transforms the inner attitude towards death, in particular countering the tendency to ignore one’s own mortality. Due to being an integral part of the description of the first establishment of mindfulness in the *Satipaṭṭhāna-sutta* and its parallels, such contemplation pertains to the category of cultivating right mindfulness as a factor of the noble eightfold path, thereby standing at the very heart of early Buddhist meditation practice and serving as an implementation of the direct path to the realization of Nirvana.

Social scientists have also been interested in how people respond when they realize the inevitability of death and dying. For example, according to terror management theory (Greenberg et al. 1986), mortality salience is a prime motivator of behavior change. Once mortality becomes salient, the range of responses varies from denial of one’s vulnerability to death, distress, anxiety, which often leads to a search for meaning in one’s life. The challenge that death and dying pose to human beings is that one cannot control or predict when and how these will occur (Greenberg et al. 1986). Anxiety and fear of death are the result, due to the fact that death can happen at any time. Fear of death can become accentuated through the influence of the media, for example through reports of terrorist attacks (Boscarino et al. 2004), increasing awareness of one’s own mortality during the current COVID-19 pandemic (Pyszczynski et al. 2021), or escalation of the current Russia-Ukraine war may trigger fears of a nuclear disaster (Dokoupil 2022).

Terror management theory (TMT) proposes that knowledge of the inevitability of death (mortality salience) and the consequent fear of dying can result in a strong motivation to reduce this anxiety (terror) by finding a way to cope with it (management). The theory implicates two mechanisms: cultural worldviews and self-esteem. Cultural worldviews are shared beliefs that structure the world by providing a feeling of order, sense, and durability. According to Greenberg et al. (1986, p. 198), by “providing a view of the world as orderly, predictable, meaningful, and permanent, culture allows for the possibility of minimizing our terror by denying our essential creatureliness (i.e., our impotence, vulnerability, and mortality). Self-esteem is the belief that one is living up to cultural standards and building a sense of personal value. Self-esteem consists of two components, (a) faith in a particular cultural drama, where human life is portrayed as important, enduring, and meaningful, and (b) the belief that one plays a significant role in that drama.

Furthermore, there is a tendency to believe that some valued aspect of oneself will continue to exist after one’s biological death, a belief which serves to help cope with death-related anxiety (Martin 1999). This can take the form of either believing in an afterlife and even immortality, or, symbolically, by way of extensions of the self (e.g., achievements in life or children). Pyszczynski et al. (1997) reasoned that it is no surprise that people devote a great deal of energy to maintaining this sense of value within their cultural worldview and are motivated to defend this perspective. According to TMT, in defense of their world view, individuals may adhere to an ideology in order to shield themselves from the paralyzing dread associated with the uncertainty of what happens when one dies (Greenberg et al. 1986).

A meta-analysis of 164 research studies found that the mortality salience hypothesis of the TMT yielded moderate effects (*r* = 0.35) on the dependent variables related to worldview and self-esteem (Burke et al. 2010). The authors concluded that the mortality salience hypothesis of TMT was robust and produced moderate to large effects when mortality salience was manipulated and thus also on attitudinal, behavioral, and cognitive dependent variables. Several studies have also shown that high self-esteem correlates with less anxiety about death, which also supports the hypotheses of TMT (Pyszczynski et al. 2004). Although there is substantial empirical support for the tenets of TMT, evolutional psychologists (e.g., Buss 1997; Kirkpatrick & Navarrete 2006; Leary & Schreindorfer 1997; Wong & Tomer 2011) have argued that fear of death is an adaptive response resulting from natural selection, without which humans would not have been able to avoid maladaptive situations. The argument is that, if human beings behave in ways that avoid situations that are likely to lead to death, then mortality salience effects reflect adaptive responses to certain life threats. The counterargument by TMT theorists is that such criticism confuses fear related to immediate and direct danger with anxiety related to thoughts of future threats which may or may not eventuate (Landau et al. 2007). Indeed, these authors have attempted to explore the compatibility of TMT theory and evolutionary psychology, which emphasize different aspects of a perceived time frame of mortality (e.g., immediate vs delayed).

A number of mortality induction studies have assessed a range of factors correlated with responses to mortality salience, including proximal and distal defenses (Pyszczynski et al. 1999). For example, Schultz and Arnau (2019) assessed the effects of brief mortality salience induction in undergraduate students across three experimental groups. They found that mindfulness and mind-wandering groups had fewer proximal defense responses when compared to the worrying group but no differences in distal defense responses across the three groups. As noted by Moon (2019), induced mindfulness of death salience may have positive psychological effects. In addition, recent studies have investigated how mindfulness may affect responses to mortality salience. For example, Askarizadeh et al. (2022) found that mindfulness in pregnant women was associated with an increase in cognitive flexibility, which in turn was associated with reduced death anxiety. Bianco et al. (2019) reported that people who had a near-death experience showed greater mindfulness than those who did not have near-death experience. Furthermore, Cacciatore et al. (2015) found that death education increased mindfulness in social work students.

Generally, theoretical and empirical evidence indicates that mindfulness may reduce fear of death and dying. However, there is lack of research examining specific effects of brief mindfulness practices daily over 6 weeks on death-related fears. The present study aimed to compare the effects of brief mindfulness practices (experimental) and contemplative practices (active control) on fear of death and dying as well as related variables, such as mindfulness facets and self-compassion. In this context, mindfulness was defined as a practice of purposely bringing one’s attention into the present moment and without judgement while contemplation was described as studying or observing something carefully or thinking deeply about something. Therefore, the mindfulness intervention and the contemplative control condition were experientially distinct types of practices. It was hypothesized that both brief mindfulness practices and contemplative practices used daily over a period of 6 weeks would reduce fear of death while increasing mindfulness and self-compassion.

## Methods

### Participants

Undergraduate psychology students were invited to participate in the study by an announcement located on the university website. Those who consented to participate in the study received 1% bonus marks for completing each instance of the survey. In other words, they received 1% bonus course marks for completing the survey at baseline, 2% for completing the survey at both baseline and post-intervention and 3% for completing the survey at baseline, post-intervention and at follow-up (1‒3 weeks following post-intervention). Initially, 103 participants completed the survey at the baseline but 14 participants were excluded from the study because they declined to participate in the intervention. Therefore, the final sample included 89 participants. Demographic characteristics of the participants are included in Table 1. All participants were undergraduate university students with a majority being females (82%) and New Zealand European (67.4%). The second largest ethnic group were Māori (14.6%), the indigenous people of New Zealand. There was no significant disproportion in the distribution of sex and ethnicity between intervention and control groups. A half of the overall sample reported meditation experience over 1 year while 22% never practiced meditation.

**Table 1:** Participants demographics

Characteristics	Overall sample	Mindfulness	Contemplation
	*n* = 89	*n* = 44	*n* = 45
Age mean (SD)	26.74 (7.76)	27.30 (7.43)	25.40 (6.85)
Age range	20–51	20–49	20–51
Females	73 (82%)	36 (82%)	37 (82%)
Ethnicity			
European	60 (67.4%)	30 (68.1%)	30 (66.6%)
Māori	13 (14.6%)	5 (11.4%)	8 (17.8%)
Pasifika	1 (1.1%)	0 (0.0%)	1 (2.2%)
Asian	7 (7.9%)	4 (9.1%)	3 (6.7%)
Other	8 (9.0%)	5 (11.4%)	3 (6.7%)
Meditation experience			
Never practiced	22 (22%)	10 (20%)	12 (24%)
< 1 year	26 (26%)	16 (32%)	10 (20%)
> 1 year	51 (51%)	23 (47%)	21 (42%)

### Procedure

The participants were randomly assigned using the block randomization method to either the mindfulness (intervention) group (*n* = 44) or the contemplation (active control) group (*n* = 45). They were provided with a different meditation or contemplation recording each week, respectively, over 6 weeks. These recordings were accessible through personal online link emailed to each participant individually. The participants were instructed to listen and complete a recorded mindfulness or contemplative practice (20 min each) each day for 6 weeks. They also had an option to use their meditation or contemplation experiences for their course work as an alternative to conducting a literature review on related topics.

#### Mindfulness Intervention

The mindfulness intervention included six recorded sessions of 20 min each on the following topics: (1) introduction to mindfulness with emphasis on whole body awareness; (2) introduction to mindfulness of breathing; (3) mindfulness of breathing with emphasis on impermanence; (4) mindfulness of breathing with emphasis on letting go; (5) mindfulness of breathing with emphasis on the significance of the breath for staying alive; and (6) mindfulness of breathing with emphasis on the fact that the breath keeps ending. However, the instructions did not explicitly bring up the topic of death and dying. These sessions were recorded by a mindfulness practitioner with extensive experience in teaching mindfulness-based practices. Participants assigned to the mindfulness intervention group were asked to complete the recorded practices daily for 6 weeks, with a different meditation each week.

#### Contemplation

The contemplative control intervention was aligned with the mindfulness intervention in terms of number and length of sessions, which were recorded by a psychologist with the relevant master degree, and covered the following topics: (1) mother nature and one’s internal landscape; (2) allowing painful thoughts and emotions, and observing them; (3) allowing mental events to dissolve, to enable change; (4) recognizing humanity in others and developing empathy; (5) acting virtuously and developing respect for oneself; and (6) experiencing anger and guilt, and letting them go. These contemplations were based on the Daily Examen described by St. Ignatius in his *Spiritual Exercises* (https://www.jesuits.org/spirituality/the-ignatian-examen/). Participants who were assigned to the contemplation control group were instructed to complete the practices daily for 6 weeks, with a different contemplation each week.

### Measures

The participants completed the following rating scales at baseline, post-intervention, and follow-up.

#### Fear of Death

The 36-item Collett–Lester Fear of Death Scale (CLFDS; Lester 1990) was developed to measure fears of death operationalized as four distinct subscales including dying of oneself, death of self, dying of others, and death of others. It uses 5-point Likert scales to assess relevant levels of anxiousness from 1 (not anxious) to 5 (very anxious). Negatively worded items are reverse coded before computing sum scores for each subscale. Computed with our data, two subscales measuring death of oneself and dying of oneself demonstrated acceptable reliability with McDonald’s omega coefficients of *ω* = 0.85 and *ω* = 0.68 respectively. Two other subscales did not converge for estimating Omega coefficients and Cronbach’s alpha for the subscale measuring fears related to the death of others was low (*α* = 0.65), while the subscale measuring fears related to the dying of others showed poor reliability (*α* = 0.56). Item 13 (“I would like to communicate with the spirit of a friend who has died”) displayed unacceptably low item-to-total correlation (0.02) and after removing this item the reliability of this subscale was improved (*ω* = 0.76). In the subscale measuring fear of dying of others, 4 items (8, 10, 11, and 35) had low item-to-total correlations (below 0.20), and after removing these items the subscale reliability was improved (*ω* = 0.67) but was still only marginally acceptable. Therefore, we used the two modified subscales which had acceptable reliability.

#### Mindfulness

The Five Facet Mindfulness Questionnaire (FFMQ; Baer et al. 2006) includes 39 items and was developed to assess these five facets of mindfulness: Observe, Describe, Acting with awareness (Act), Nonreactivity to inner experience (Nonreact), and Nonjudging of inner experience (Nonjudge). Research suggests that the FFMQ has acceptable psychometric properties and its subscales measure relatively stable individual characteristics or traits (Truong et al. 2020). The scale uses a 5-point Likert scale format ranging from 1 (never or very rarely true) to 5 (very often or always true) with higher scores reflecting higher levels of mindfulness. There are 19 negatively worded items that require reverse coding prior to computing subscales scores. After these items are reverse coded, subscales scores are computed by adding items scores together. All subscales showed good reliability with the current dataset using McDonalds Omega estimate: Observe (*ω* = 0.87), Describe (*ω* = 0.93), Act (*ω* = 0.90), Nonjudge (*ω* = 0.93), and Nonreact (*ω* = 0.89).

#### Self-compassion

The Self-Compassion Scale-Short Form (SCS-SF; Raes et al. 2011) includes 12 items psychometrically selected from the original 26-item scale. It covers six aspects of self-compassion including self-kindness (e.g., "I try to be understanding and patient towards those aspects of my personality I don’t like"), self-judgement (e.g., "I’m disapproving and judgemental about my own flaws and inadequacies"), common humanity (e.g., "I try to see my failings as part of the human condition"), isolation (e.g., "When I’m feeling down, I tend to feel like most other people are probably happier than I am"), mindfulness (e.g., "When something upsets me I try to keep my emotions in balance"), and over-identification (e.g., "When I fail at something important to me I become consumed by feelings of inadequacy") (Neff 2003, p. 231). Respondents indicate the extent to which each statement representing the scale item is true for them using a 5-point Likert scale format ranging from 1 (almost always) to 5 (almost never). There are six negatively worded items: two items per domain including self-judgement, isolation, and over-identification. These items are reverse coded to ensure that higher scores reflect higher levels of self-compassion on the scale. The scale provides a valid total self-compassion score while use of subscales scores was not recommended for the SCS-SF due to reliability issues (Finaulahi et al. 2021; Medvedev et al. 2021a). The SCS-SF demonstrated excellent reliability with our dataset (*ω* = 0.89).

### Data Analyses

Data analyses were conducted with IBM SPSS 27. Outcome variables were analyzed using mixed model ANOVA (2 × 3) with two groups (mindfulness and contemplation) as between-subject factors and pre-intervention (baseline), post-intervention (6 weeks), and follow up (1‒3 weeks post-intervention) as within-subject factors. Post hoc tests were used to determine statistical significance of differences between groups and within groups over time if significant main effects were indicated by the omnibus ANOVA. Statistical significance was determined based on the conventional cut-off point for *p*-value = 0.05 throughout. To achieve statistical power of 80% to detect a small effect size of 0.25 under *p* = 0.05 with two groups, the minimum required sample size is 86 participants.

## Results

**Fig. 1:**
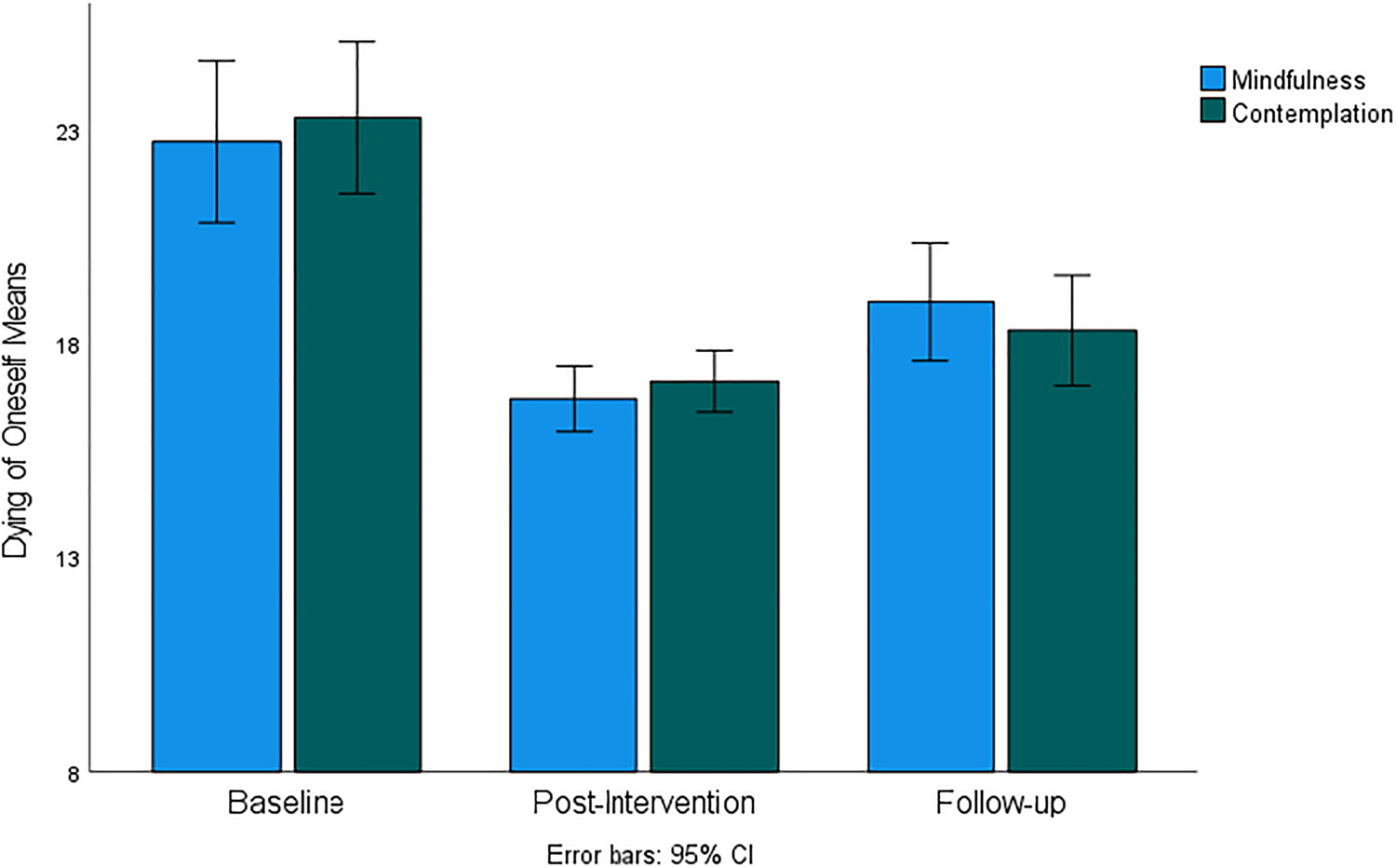
Fear of *Dying of Oneself* subscale mean scores for mindfulness and contemplation groups

Figure 1 shows a comparable decrease of fear related to the dying of oneself in both the mindfulness and the contemplation groups following intervention and at follow-up. ANOVA indicated statistically significant and large effect of time (*F*(60, 1) = 71.14, *p* < 0.001, *η*^2^ = 0.54) with no significant differences between groups at any time point and no group-time interaction suggesting that both interventions were equally effective at reducing fear associated with the dying of oneself. Post hoc tests demonstrated that mean scores of fear of oneself dying in both groups post-intervention and at follow-up were significantly lower compared to the baseline, whereas post-intervention mean scores were significantly lower compared to follow-up scores.

**Fig. 2:**
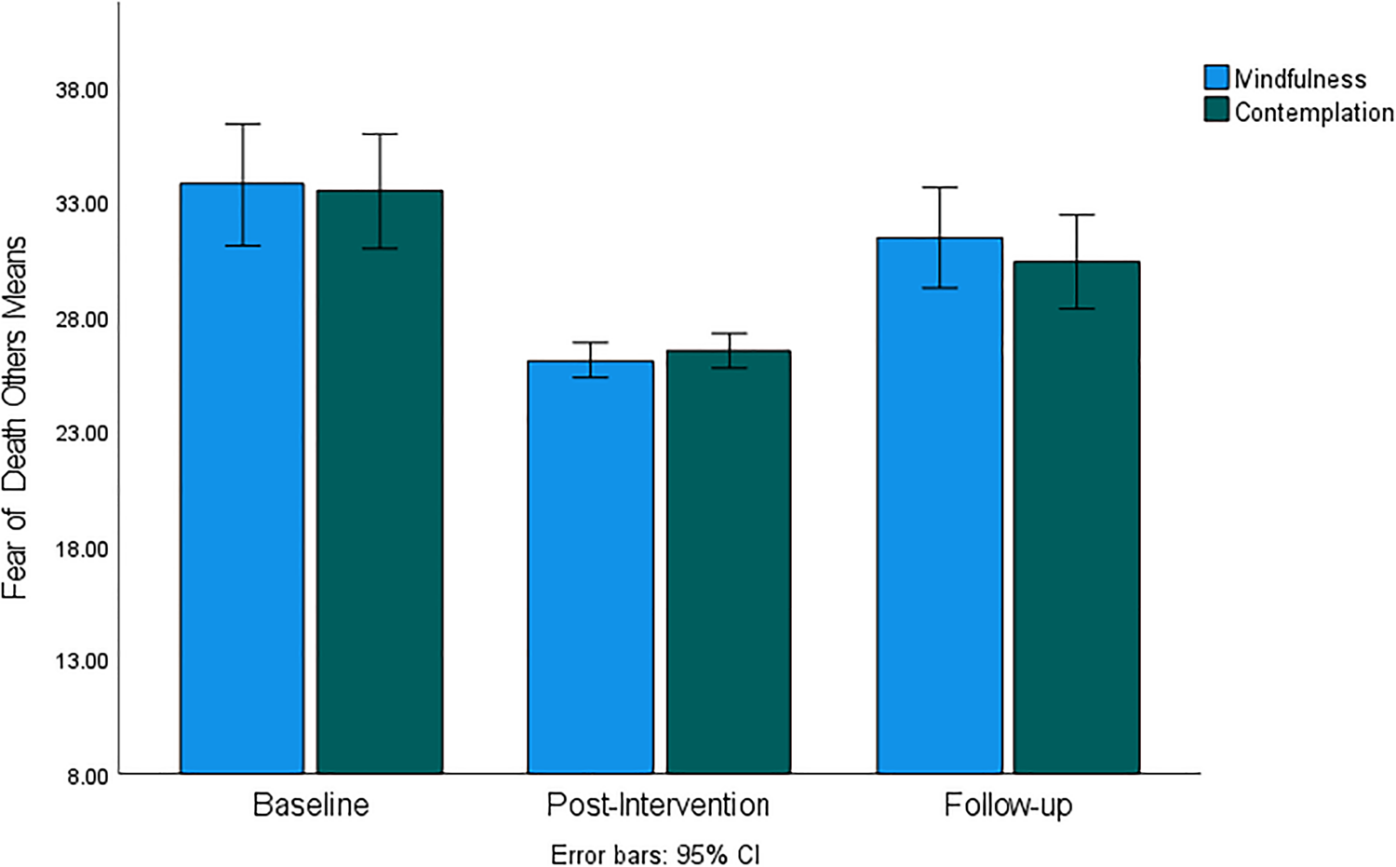
Fear of *Death of Others* subscale mean scores for mindfulness and contemplation groups

Figure 2 illustrates a large decrease in fears related to the death of others immediately after the intervention, followed by an increase at follow-up that was still below the baseline levels in both groups. There was a significant large effect of time (*F*(60, 1) = 62.29, *p* < 0.001, *η*^2^ = 0.51) but no significant group effect and no group-time interaction was revealed by ANOVA. Post hoc tests confirmed that mean scores of fear of the death of others in both groups were higher at baseline compared to both post-intervention and at follow-up while post-intervention mean scores were significantly lower compared to follow-up scores. These results confirm that both interventions were comparable in reducing fears related to the death of others.

**Fig. 3:**
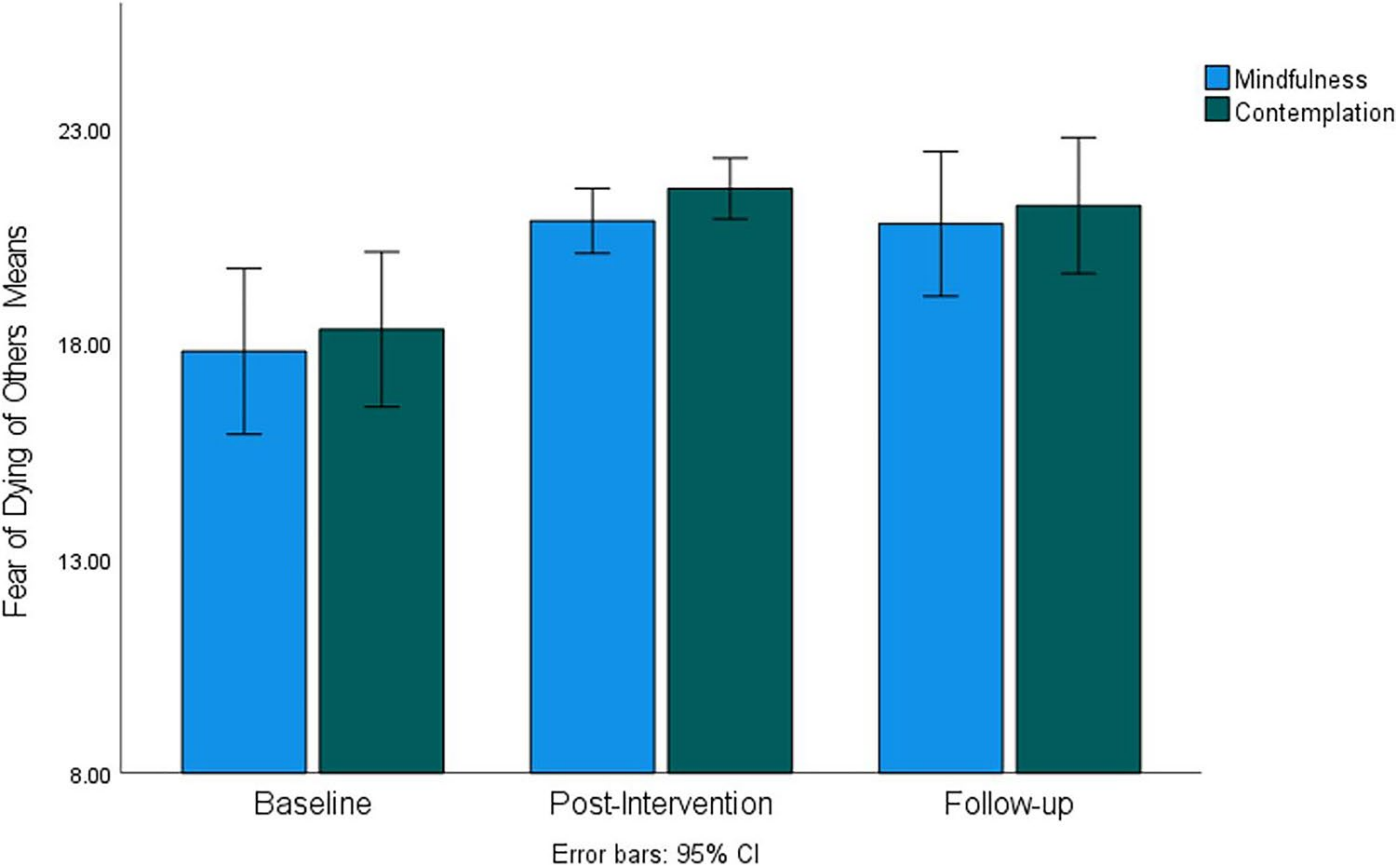
Fear of *Dying of Others* subscale mean scores for mindfulness and contemplation groups

Mean scores for fears regarding the dying of others are presented in Fig. 3 showing a noticeable increase of these fears at post-intervention with no apparent difference between post-intervention and follow-up. ANOVA found a statistically significant and large effect of time (*F*(60, 1) = 20.53, *p* < 0.001, *η*^2^ = 0.26) with no significant group effect and no interaction between time and group. Fears regarding the dying of others were significantly lower at baseline compared to both post-intervention and follow-up, but there was no significant difference between post-intervention and follow-up scores. No significant effects of time, group, or their interaction were found on fear regarding the death of oneself. Table 2 shows the descriptive statistics of all study variables including means and SDs for each group at baseline, post-intervention, and follow-up, and the *p*-values for group comparisons at each time point by independent *t*-tests. There were no significant differences between groups at any time point except for the Describe facet of the FFMQ at baseline. Overall these results indicate that mindfulness and contemplation intervention effects on all outcome variables were comparable.

**Table 2:** Descriptive statistics for all study variables including *t* test comparisons between groups

Variable	Intervention	Time	*n*	M	SD	*t* test, *p*
FFMQ Observe	Mindfulness	Baseline	44	27.14	5.56	0.386
	Contemplation	Baseline	45	28.22	6.17	0.385
FFMQ Describe	Mindfulness	Baseline	44	26.82	6.83	0.002
	Contemplation	Baseline	45	30.78	5.00	0.003
FFMQ Act	Mindfulness	Baseline	44	23.11	5.72	0.068
	Contemplation	Baseline	45	25.20	4.92	0.069
FFMQ Nonjudge	Mindfulness	Baseline	44	24.82	7.52	0.792
	Contemplation	Baseline	45	25.20	6.01	0.792
FFMQ Nonreact	Mindfulness	Baseline	44	20.93	5.37	0.404
	Contemplation	Baseline	45	21.80	4.36	0.405
Death of Oneself	Mindfulness	Baseline	44	32.84	10.19	0.625
	Contemplation	Baseline	45	33.93	10.79	0.625
Death of Others	Mindfulness	Baseline	44	41.95	6.68	0.749
	Contemplation	Baseline	45	41.44	8.23	0.749
Oneself Dying	Mindfulness	Baseline	44	22.23	5.73	0.630
	Contemplation	Baseline	45	22.78	5.00	0.631
Others Dying	Mindfulness	Baseline	44	26.48	5.07	0.572
	Contemplation	Baseline	45	25.80	6.13	0.571
Self-Compassion	Mindfulness	Baseline	44	20.20	6.29	0.803
	Contemplation	Baseline	45	20.51	5.22	0.803
FFMQ Observe	Mindfulness	Post-intervention	34	27.62	5.33	0.278
	Contemplation	Post-intervention	34	29.15	6.18	0.278
FFMQ Describe	Mindfulness	Post-intervention	34	27.65	6.49	0.008
	Contemplation	Post-intervention	34	31.41	4.80	0.009
FFMQ Act	Mindfulness	Post-intervention	34	24.59	5.71	0.209
	Contemplation	Post-intervention	34	26.18	4.55	0.209
FFMQ Nonjudge	Mindfulness	Post-intervention	34	27.56	7.42	0.576
	Contemplation	Post-intervention	34	26.62	6.36	0.576
FFMQ Nonreact	Mindfulness	Post-intervention	34	22.65	4.41	0.844
	Contemplation	Post-intervention	34	22.44	4.19	0.844
Death of Oneself	Mindfulness	Post-intervention	34	33.62	3.45	0.351
	Contemplation	Post-intervention	34	32.88	2.99	0.351
Death of Others	Mindfulness	Post-intervention	34	33.41	2.41	0.637
	Contemplation	Post-intervention	34	33.12	2.69	0.637
Oneself Dying	Mindfulness	Post-intervention	34	16.85	1.97	0.632
	Contemplation	Post-intervention	34	17.09	2.07	0.632
Others Dying	Mindfulness	Post-intervention	34	32.62	3.73	0.999
	Contemplation	Post-intervention	34	32.62	3.85	0.999
Self-Compassion	Mindfulness	Post-intervention	32	21.56	5.64	0.660
	Contemplation	Post-intervention	33	22.12	4.50	0.661
FFMQ Observe	Mindfulness	Follow-up	31	28.48	5.70	0.363
	Contemplation	Follow-up	34	29.91	6.76	0.359
FFMQ Describe	Mindfulness	Follow-up	31	29.55	5.89	0.024
	Contemplation	Follow-up	34	32.65	4.89	0.025
FFMQ Act	Mindfulness	Follow-up	31	24.61	5.85	0.052
	Contemplation	Follow-up	34	27.32	5.18	0.053
FFMQ Nonjudge	Mindfulness	Follow-up	31	27.84	8.61	0.862
	Contemplation	Follow-up	34	27.50	7.03	0.863
FFMQ Nonreact	Mindfulness	Follow-up	31	23.39	5.09	0.997
	Contemplation	Follow-up	34	23.38	4.86	0.997
Death of Oneself	Mindfulness	Follow-up	31	32.45	10.21	0.923
	Contemplation	Follow-up	34	32.71	10.97	0.923
Death of Others	Mindfulness	Follow-up	31	40.06	6.42	0.288
	Contemplation	Follow-up	34	38.29	6.86	0.287
Oneself Dying	Mindfulness	Follow-up	33	18.30	3.58	0.814
	Contemplation	Follow-up	35	18.09	3.99	0.813
Others Dying	Mindfulness	Follow-up	33	31.12	5.83	0.684
	Contemplation	Follow-up	35	31.77	7.18	0.682
Self-Compassion	Mindfulness	Follow-up	44	15.75	11.07	0.399
	Contemplation	Follow-up	45	17.73	11.00	0.399

**Fig. 4:**
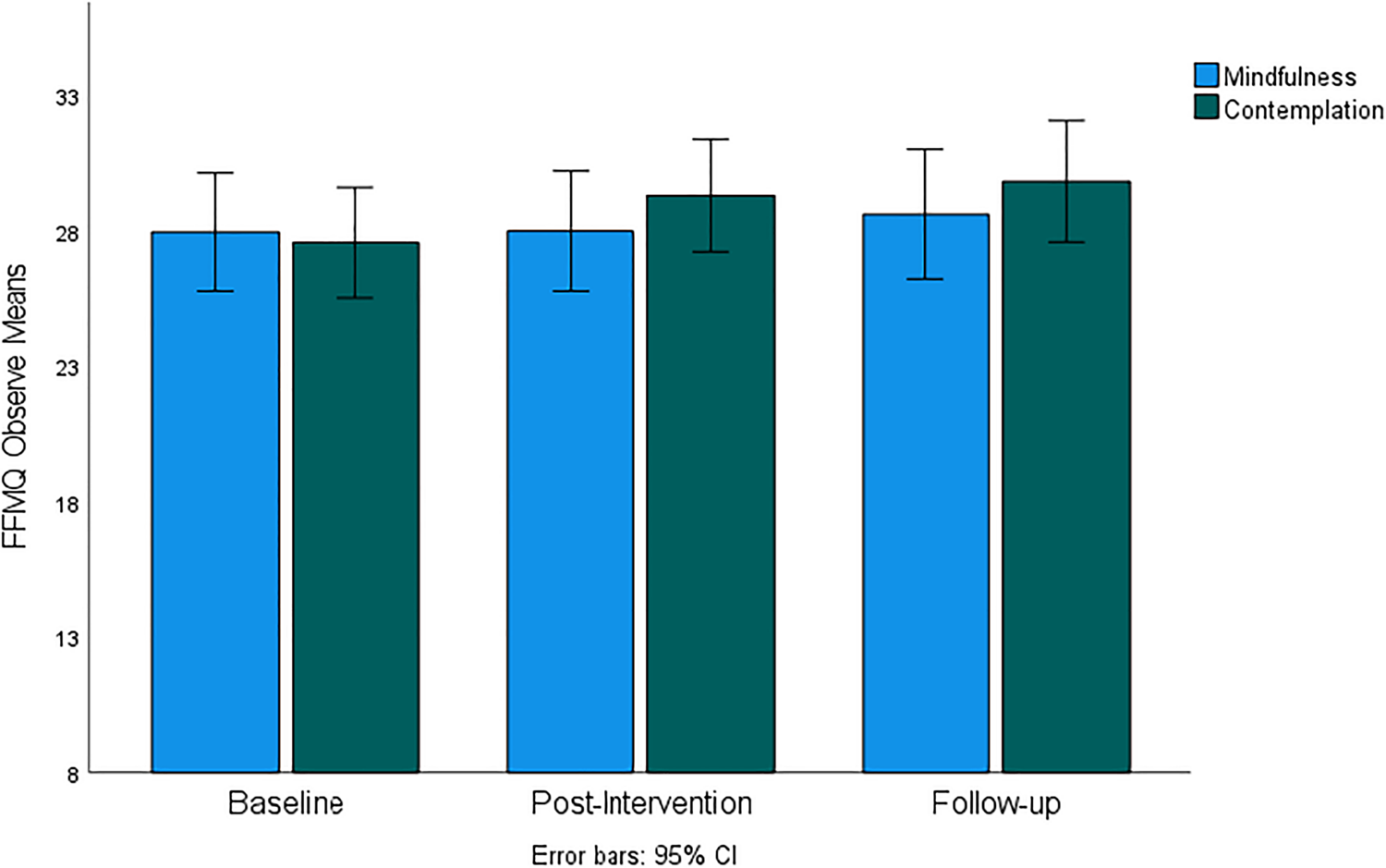
FFMQ Observe subscale mean scores for mindfulness and contemplation groups

**Fig. 5:**
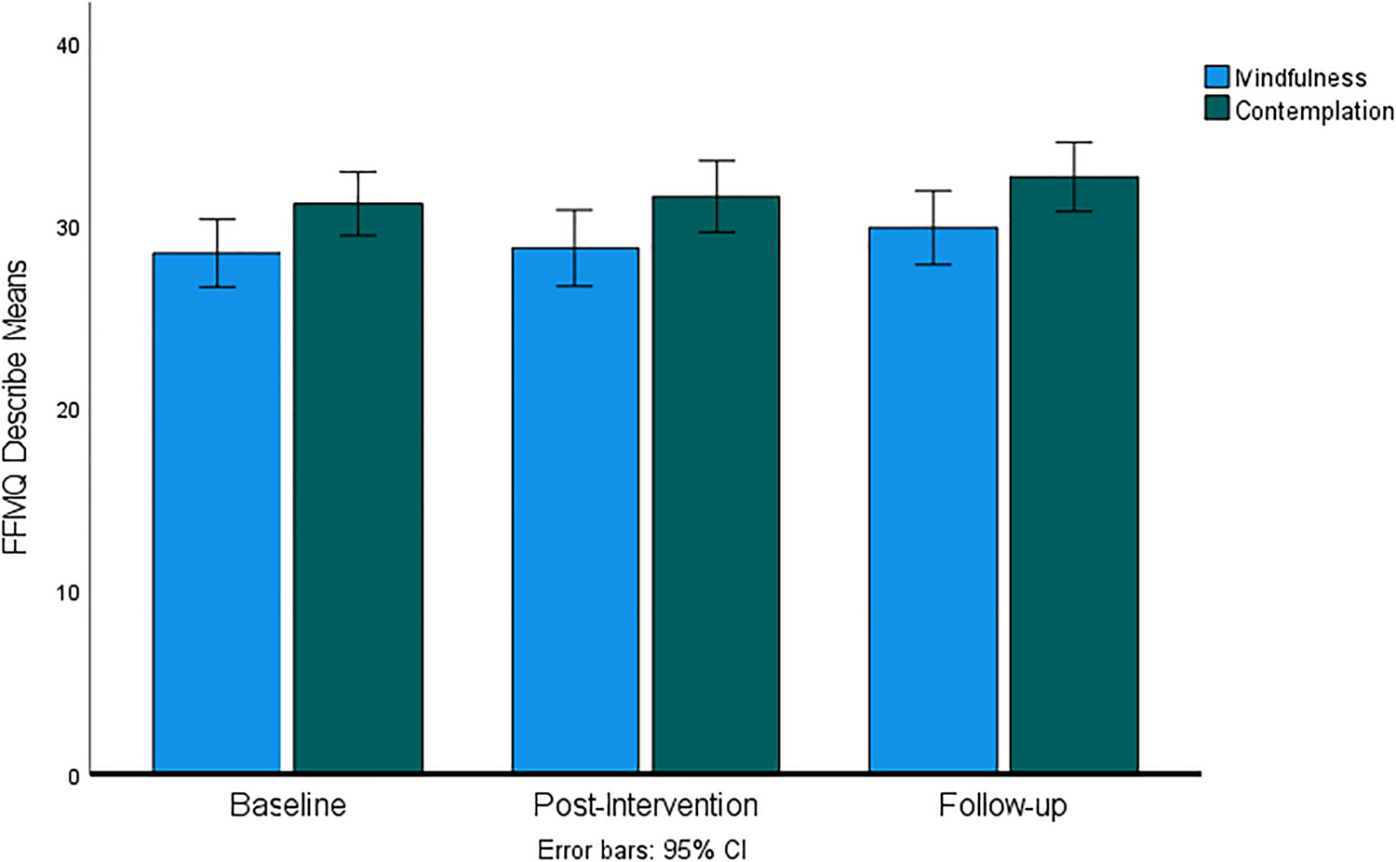
FFMQ Describe subscale mean scores for mindfulness and contemplation groups

**Fig. 6:**
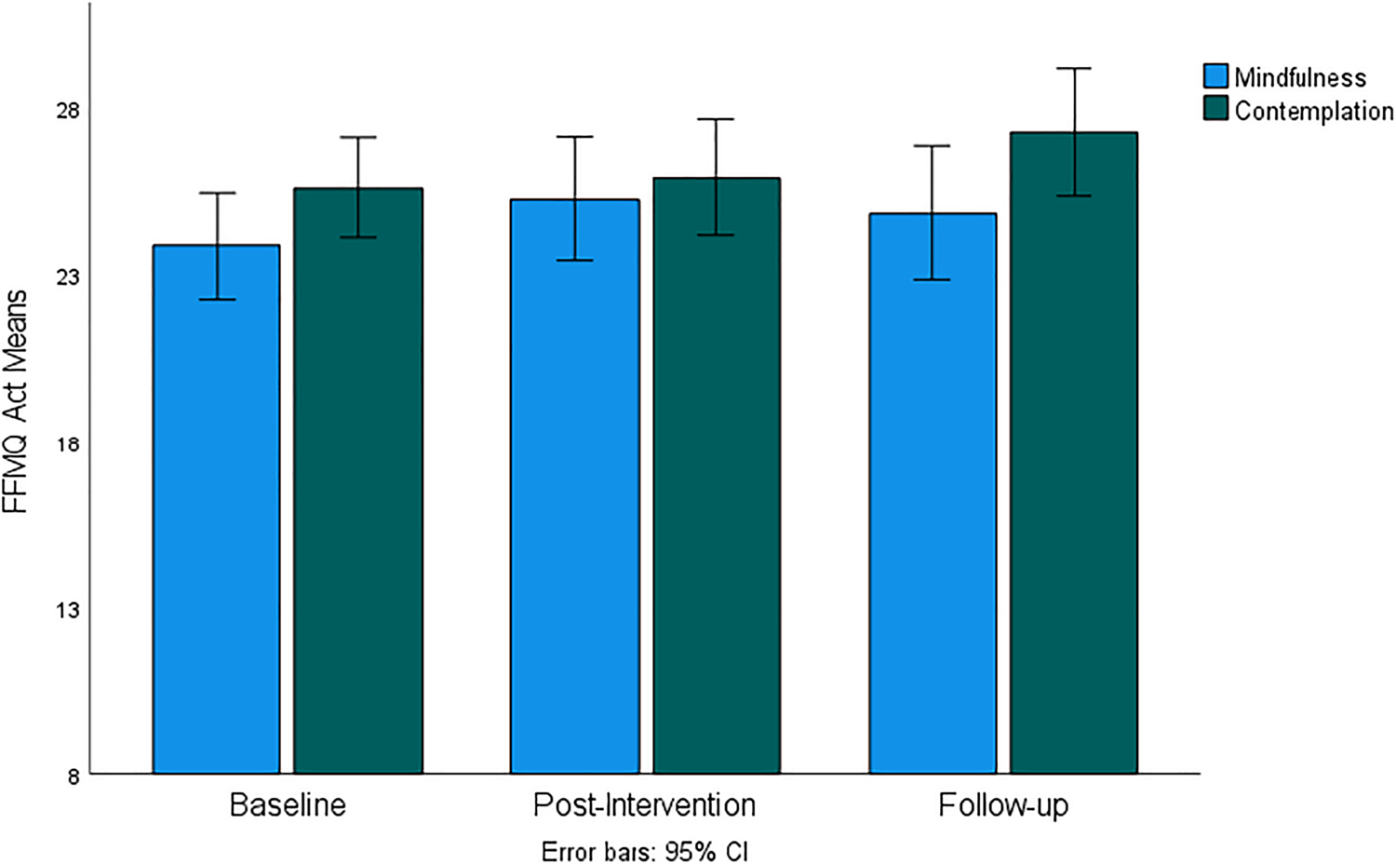
FFMQ Act subscale mean scores for mindfulness and contemplation groups

**Fig. 7:**
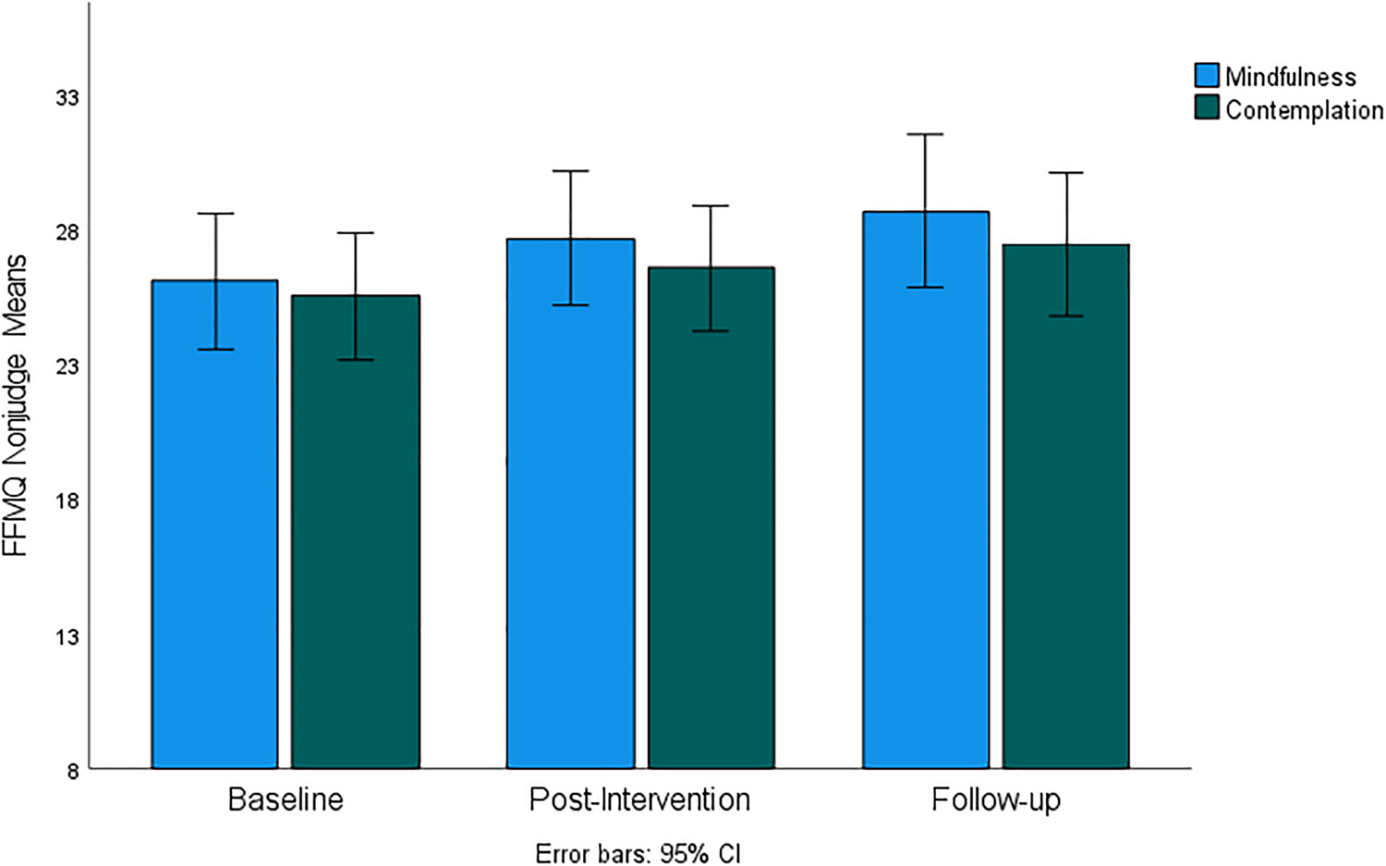
FFMQ Nonjudge subscale mean scores for mindfulness and contemplation groups

**Fig. 8:**
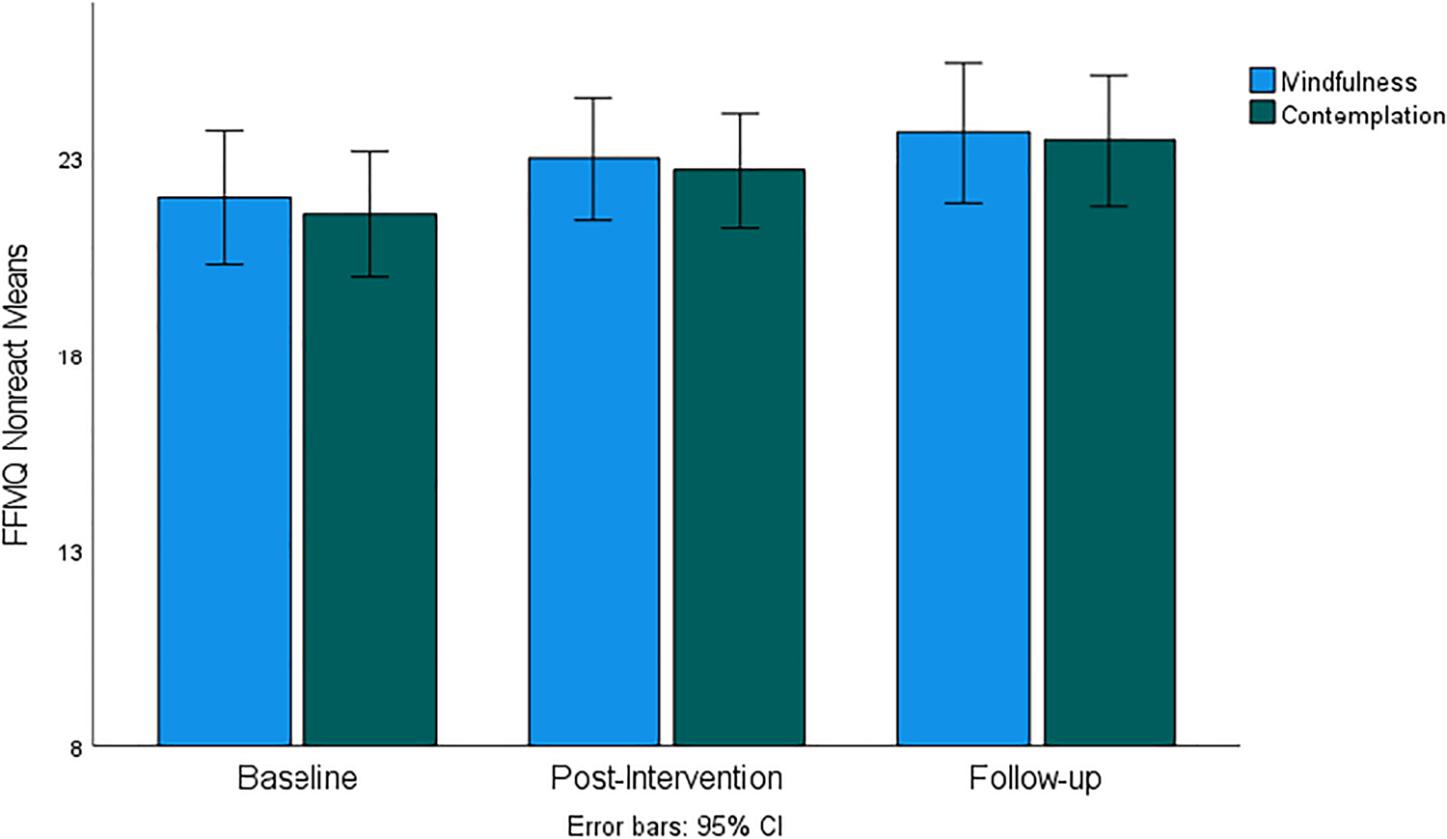
FFMQ Nonreact subscale mean scores for mindfulness and contemplation groups

Figures 4, 5, 6, 7, and 8 demonstrate a uniform increase in the mindfulness facets mean scores over time, which vary slightly across facets. ANOVA supported this trend by indicating statistically significant effect of time for the Observe facet (*F*(60, 1) = 4.45, *p* < 0.014, *η*^2^ = 0.07), Describe (*F*(60, 1) = 5.45, *p* < 0.007, *η*^2^ = 0.08), Act (*F*(60, 1) = 5.10, *p* < 0.007, *η*^2^ = 0.08), Nonjudge (*F*(60, 1) = 8.16, *p* < 0.001, *η*^2^ = 0.12), and Nonreact (*F*(60, 1) = 11.03, *p* < 0.001, *η*^2^ = 0.16). For the Nonjudge and Nonreact facets, differences between baseline, post intervention, and follow-up were all statistically significant as verified by post hoc tests (all *p*-values < 0.05). These findings suggest that an increase in nonreactivity and a non-judgemental attitude continued after intervention. However, post hoc tests also indicated that increased scores on the Observe, Describe, and Act facets were only statistically significant between baseline and follow-up but not at post-intervention. No significant effects of group and interaction between group and time were found for any of the mindfulness facets.

**Fig. 9:**
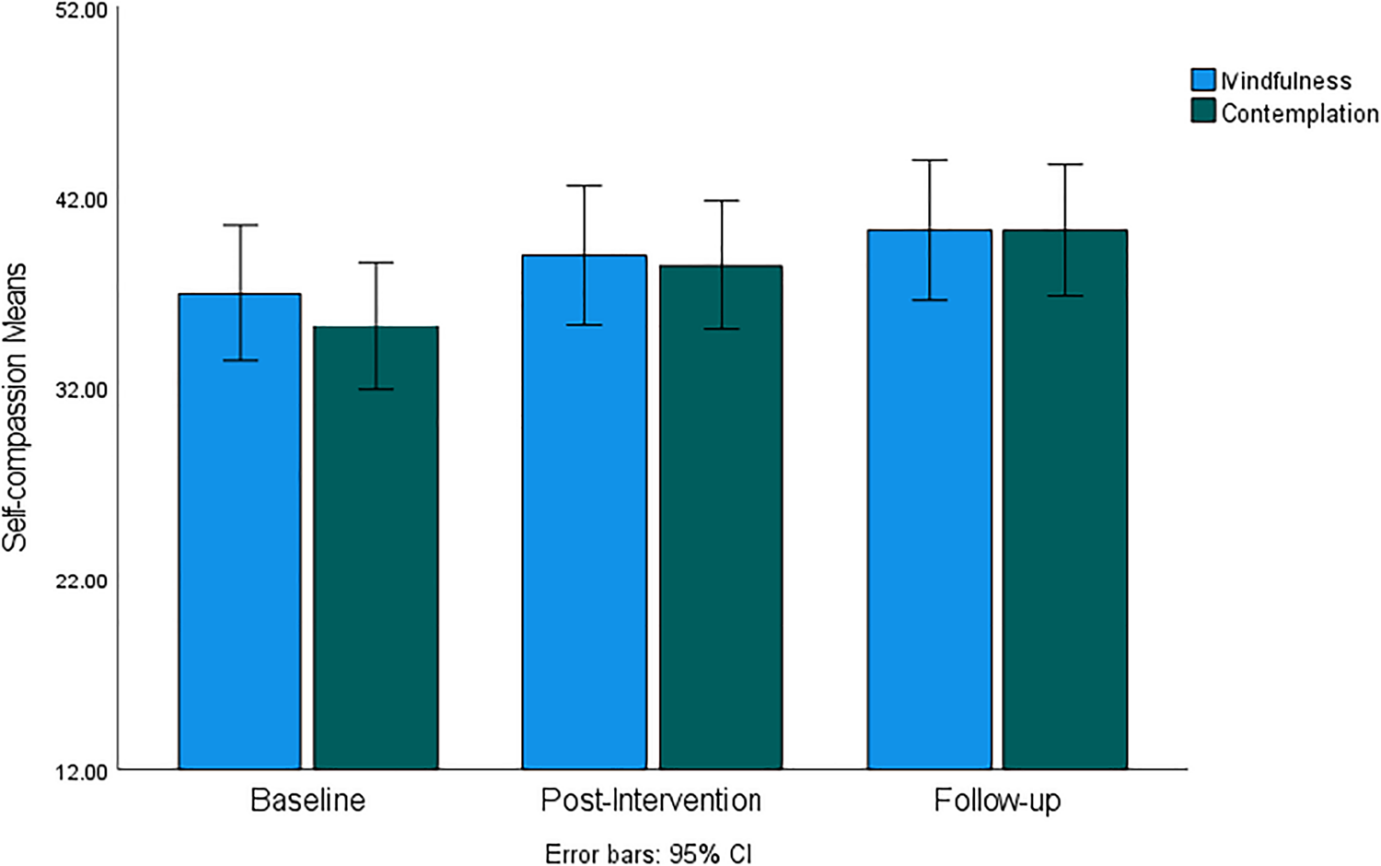
Self-Compassion mean scores for mindfulness and contemplation groups

Similar to the mindfulness facets, there was a statistically significant increase in self-compassion over time (*F*(60, 1) = 14.24, *p* < 0.001, *η*^2^ = 0.19), which is illustrated in Fig. 9. Differences between baseline and post-intervention and between post-intervention and follow-up were statistically significant, indicating that effects of both the mindfulness and contemplation interventions continued after both interventions were completed at post-intervention. However, there were no significant group-time interactions and group effects observable, which suggests a comparable effectiveness of both the mindfulness and the contemplation practices to increase self-compassion in this sample population.

## Discussion

We investigated the effects of brief mindfulness and contemplative practices completed over 6 weeks on the fear of death and dying. The results demonstrated that both mindfulness and contemplative practices significantly reduced fear of oneself dying and fear of the death of others. At the same time, significant increases on all five mindfulness facets and self-compassion were evident in both groups, which were most prominent for a non-judgemental attitude, nonreactivity, and self-compassion. Interestingly, in both groups we also found a significant increase in fear related to the dying of others. However, the effects of the mindfulness and contemplative practices on fears related to the death of oneself did not reach statistical significance. These findings may have potential implications for reducing fear of death while increasing mindfulness and self-compassion through the use of both brief mindfulness and contemplative practices but await replication.

The mindfulness practices were designed to assist the participants to develop mindfulness skills that would enable them to counter anxiety related to impermanence. The contemplative practices (the active control condition) were designed to provide a conceptually parallel program to mindfulness, which theoretically should have an equal probability of being effective. Indeed, the results showed the two interventions were generally equally effective. Upon closer examination, it is apparent that three items in the contemplative practices overlapped substantially with mindfulness practices, if viewed from a Buddhist perspective. These three items included the following: allowing for painful thoughts and emotions, and observing them; allowing for mental events to dissolve to allow for change; and experiencing anger and guilt, and letting them go. The close approximation that these three items have with mindfulness practice means that the two conditions were not clearly distinct from each other conceptually and may not have provided a true differential test of the effectiveness of the two practices.

The FFMQ was used to measure trait mindfulness of the participants at baseline, post-intervention, and at follow-up. The results showed that the FFMQ captured changes in mindfulness across experimental phases. Two facets (i.e., nonreactivity and a non-judgemental attitude) were found to increase at all three timepoints at statistically significant levels. The remaining three facets (observe, describe, and act) also increased at all three timepoints but the increases were statistically significant only between baseline and follow-up, not at post-intervention. These findings are generally consistent with the literature indicating that non-judgemental attitude and nonreactivity are stronger inverse predictors of affective symptoms, including fear and anxiety (Medvedev et al. 2018, 2021b).

In TMT (Terror Management Theory), self-esteem is considered one of the basic defense mechanisms that help people cope with death anxiety. However, it has been considered somewhat inefficient with respect to dealing with the purported psychological effects of the terror of death (Baumeister et al. 2003). For example, high self-esteem has been reported to be linked to self-enhancement bias, which may lead participants to believe the superiority of their own group when compared to other groups (Sedikides & Gregg 2008), or to judge themselves as superior to their in-group peers (Alicke & Govorun 2005). These findings have led researchers to investigate whether self-compassion may be a more positive adaptive response to terror management with regard to death anxiety. For example, in a mortality priming study with college students, Gerber and Anaki (2019) reported that self-compassion was associated with avoidance of concrete threats (e.g., images of people with hostile expressions) but not with abstract threats (e.g., images of people with physical injury). Furthermore, given the use of Neff’s (2003) self-compassion scale, it was found that the common humanity but not the mindfulness sub-scale was the buffering defense mechanism against concrete threats. The fact that there was an increase in self-compassion in the present study due to the mindfulness and the contemplative practices suggests that these interventions may be targeting a key variable that could result in clinical improvements in people facing existential threats in their lives. While there is reasonably robust evidence indicating that mindfulness-based programs (such as Mindfulness-based Stress Reduction, Kabat-Zinn 1990) enhance self-compassion (Wasson et al. 2020), the relatively brief mindfulness intervention used in the present study may also be clinically useful.

Recent research has begun to focus on mindfulness as a correlate, predictor, and intervention for a person’s fear of death (Moon 2019; Niemiec et al. 2010). Mortality salience can be enhanced in different ways. In the Buddhist tradition, cultivation of recollection of one’s passing away even with the next breath is a mindfulness practice (Anālayo 2016, 2020). In experimental research, this would require programming the use of mindfulness specifically for recollection of death within the intervention program. However, due to ethical constraints, the instructions did not explicitly raise the topic of death and dying in the present study. As a result, the mindfulness intervention did not include an actual mindfulness of death practice. It included only some implicit pointers in that direction but did not address the topic explicitly. Future research will need to develop a suitable experimental paradigm to assess the scientific veracity of this Buddhist teaching and its implications for mindfulness and other contemplative interventions. It is imperative that humans have explicit knowledge of and instructions on mindfulness of death, should they choose to engage in its practice, because death may occur at any time.

The present study was undertaken during the COVID-19 pandemic, a time of greater awareness of death and dying. That is, the pandemic most likely increased mortality salience over a prolonged period. In terms of TMT, the pandemic resulted in heightened anxiety and existential terror of death in many people (Pyszczynski et al. 2021). How individual participants in the present study differentially reacted to the pandemic is an open question. Indeed, college students in the age range of the participants in the present study have reported a sharp increase in anxiety related to health issues during the pandemic (Hoyt et al. 2021), as well as concerns about their own health and those of their family members (Hagedorn et al. 2022). Taking into account earlier findings that women generally self-report higher death anxiety than men (Lester et al. 2007), and that non-white participants report higher levels of death anxiety than their white peers (Chow 2017), it is critical that these findings be used as benchmarks when assessing the strength of the present findings. Future research needs to tease out the percentage of variance that can be accounted for by these variables when assessing the effects of mindfulness and contemplative practices for facing the terror of mortality.

### Limitations and Future Research

The outcomes of the present research should be contextualized within the limitations of our study. The study included two arms, intervention and an active control, both of which were effective in a similar manner. While using an active control condition is a strength of this study, it did not enable us to determine if either intervention is better than a no-intervention condition. Although highly unlikely due to the length of the interventions, theoretically it could be argued that the observed changes may have occurred due to history or other non-specific intervening variables that coincided with the experimental and active control conditions. Thus, the findings from the present study need to be replicated in a three-arm trial with a non-intervention control condition.

The two interventions were matched on a number of variables, including the number of training sessions, session length, and total training time. The audio recordings of the instructions for both interventions were recorded by skilled and experienced meditation and contemplation instructors. The only possible confound is that two instructors were used, one who was a specialist in mindfulness and the other who was an expert in contemplative practices. Future research may wish to assess if instructor differences in terms of vocal qualities may have played any role in the outcomes. For example, could any one or a combination of volume, pace, pitch, rate, rhythm, fluency, articulation, pronunciation, enunciation, tone, and emotion have had an impact? Or, perhaps a suitable instructor can be used who has extensive experience in both the experimental and active control interventions.

The overall sample was not representative of the broader population of New Zealand because it was skewed towards women, which may limit generalizability. While the 2018 New Zealand census reported the sex ratio to be 0.97 males/females, 82% of our sample were females. Furthermore, given that the sample was from the University of Waikato, at which 58.7% and 62.7% of the total and graduate enrollments are females, respectively, the ratio of females in our sample was higher than that in the country and at the participants’ university. However, the racial composition was much closer to being representative of the country, with 67% being of European origins (census: 71.8%) and 14.6% being Māori (census: 16.5%). Unless participant characteristics are stratified by design, they are unpredictable when there is an open call for study subjects on the Internet. Another aspect of the study sample, that the participants were self-selected, may limit the generalizability of the findings to university students generally or even at the same university.

When multiple constructs (e.g., fear of death, mindfulness, self-compassion) are rated using common methods (e.g., within the same Internet survey), spurious effects may result due to the measurement instruments rather than to the constructs being measured. Our study may have suffered from common method bias (Podsakoff et al. 2012), which has been reported in research using multiple self-reported measures. These effects may occur as a result of specific response styles, social desirability, and priming effects that are distinct from the true associations among constructs. Future research on the mindfulness management of the terror of mortality could ensure that the same participants are measured on multiple constructs using multiple methods or instruments (Bagozzi & Yi 1993).

## Supplementary Information

Below is the link to the electronic supplementary material.

Supplementary file1 (XLSX 28 KB)

## Data Availability

The online version contains de-identified data for this study in Supplementary File S1.

## Abbreviations

AN: *Aṅguttara-nikāya*
EĀ: *Ekottarika-āgama* (T 125)
MĀ: *Madhyama-āgama* (T 26)
MN: *Majjhima-nikāya*
SĀ: *Saṃyukta-āgama* (T 99)
SN: *Saṃyutta-nikāya*
T: Taishō edition

## References

[CR1] Alicke MD, Govorun FO, Alicke MD, Dunning DA, Krueger J (2005). The better-than-average effect. The self in social judgment.

[CR2] Anālayo, Bh. (2013). *Perspectives on satipaṭṭhāna*. Windhorse Publications.

[CR3] Anālayo Bh (2014). The Buddha’s last meditation in the Dīrgha-āgama. Indian International Journal of Buddhist Studies.

[CR4] Anālayo, Bh. (2016). *Mindfully facing disease and death, compassionate advice from early Buddhist texts*. Windhorse Publications.

[CR5] Anālayo, Bh. (2017). *A meditator’s life of the Buddha, based on the early discourses*. Windhorse Publications.

[CR6] Anālayo, Bh. (2020). *Mindfulness in early Buddhism, characteristics and functions*. Windhorse Publications.

[CR7] Anālayo, Bh. (2022a). Recollection in early Buddhist meditation. In N. N. Singh (Ed.), *Encyclopedia of mindfulness, Buddhism, and other contemplative practices*. Springer. 10.1007/978-3-030-90465-4_53-1.

[CR8] Anālayo Bh (2022). Visualization in early Buddhism. Mindfulness.

[CR9] Askarizadeh G, Poormirzaei M, Bagheri M (2022). Mindfulness facets and death anxiety: The role of cognitive flexibility components. Psychological Studies.

[CR10] Baer RA, Smith GT, Hopkins J, Krietemeyer J, Toney L (2006). Using self-report assessment methods to explore facets of mindfulness. Assessment.

[CR11] Bagozzi RP, Yi Y (1993). Multitrait-multimethod matrices in consumer research: Critique and new developments. Journal of Consumer Psychology.

[CR12] Baumeister RF, Campbell JD, Krueger JI, Vohs KD (2003). Does high self-esteem cause better performance, interpersonal success, happiness, or healthier lifestyles?. Psychological Science in the Public Interest.

[CR13] Bianco, S., Testoni, I., Palmieri, A., Solomon, S., & Hart, J. (2019). The psychological correlates of decreased death anxiety after a near-death experience: The role of self-esteem, mindfulness, and death representations. *Journal of Humanistic Psychology*. Advance of Print. 10.1177/0022167819892107

[CR14] Boscarino JA, Figley CR, Adams RE (2004). Compassion fatigue following the September 11 terrorist attacks: A study of secondary trauma among New York City social workers. International Journal of Emergency Mental Health.

[CR15] Brooks T (2005). Better luck next time: A comparative analysis of Socrates and Mahāyāna Buddhism on reincarnation. Journal of Indian Philosophy and Religion.

[CR16] Burke BL, Martens A, Faucher EH (2010). Two decades of terror management theory: A meta-analysis of mortality salience research. Personality and Social Psychology Review.

[CR17] Buss DM (1997). Human social motivation in evolutionary perspective: Grounding terror management theory. Psychological Inquiry.

[CR18] Cacciatore J, Thieleman K, Killian M, Tavasolli K (2015). Braving human suffering: Death education and its relationship to empathy and mindfulness. Social Work Education.

[CR19] Chow HPH (2017). A time to be born and a time to die: Exploring the determinants of death anxiety among university students in a western Canadian city. Death Studies.

[CR20] Dokoupil, T. (2022, April 29). Conflict in Ukraine triggers fear of nuclear warfare*.* CBS Interactive Inc. https://www.cbsnews.com/news/conflict-nuclear-warfare-ukraine-russia/

[CR21] Fernández-Campos S, Roca P, Yaden MB (2021). The impermanence awareness and acceptance scale. Mindfulness.

[CR22] Finaulahi KP, Sumich A, Heym N, Medvedev ON (2021). Investigating psychometric properties of the Self-Compassion Scale using Rasch methodology. Mindfulness.

[CR23] Gerber Z, Anaki D (2019). Self-compassion as a buffer against concrete but not abstract threat. Death Studies.

[CR24] Greenberg, J., Pyszczynski, T., & Solomon, S. (1986). The causes and consequences of a need for self-esteem: a terror management theory. In R. F. Baumeister (Ed.), *Public self and private self* (pp. 189‒212). Springer, 10.1007/978-1-4613-9564-5_10.

[CR25] Hagedorn RL, Wattick RA, Olfert MD (2022). “My entire world stopped”: College students’ psychosocial and academic frustrations during the COVID-19 pandemic. Applied Research in Quality of Life.

[CR26] Hoyt LT, Cohen AK, Dull B, Castro EM, Yazdani N (2021). Constant stress has become the new normal: Stress and anxiety inequalities among US college students in the time of COVID-19. Journal of Adolescent Health.

[CR27] Kabat-Zinn J (1990). Full catastrophe living: Using the wisdom of your body and mind to face stress, pain, and illness.

[CR28] Kirkpatrick LA, Navarrete CD (2006). Reports of my death anxiety have been greatly exaggerated: A critique of terror management theory from an evolutionary perspective. Psychological Inquiry.

[CR29] Kumoi S (1969). Der Nirvāṇa-Begriff in den kanonischen Texten des Frühbuddhismus. Wiener Zeitschrift Für Die Kunde Südasiens.

[CR30] Landau, M. J., Solomon, S., Pyszczynski, T., & Greenberg, J. (2007). On the compatibility of terror management theory and perspectives on human evolution. *Evolutionary Psychology*, *5*(3), 10.1177/147470490700500303.

[CR31] Leary MR, Schreindorfer LS (1997). Unresolved issues with terror management theory. Psychological Inquiry.

[CR32] Lester D (1990). The Collett-Lester fear of death scale: The original version and a revision. Death Studies.

[CR33] Lester D, Templer DI, Abdel-Khalek A (2007). A cross-cultural comparison of death anxiety: A brief note. Omega: Journal of Death and Dying.

[CR34] Martin, L. L. (1999). ID compensation theory: Some implications of trying to satisfy immediate-return needs in a delayed-return culture. *Psychological Inquiry, 10*(3), 195‒208. http://www.jstor.org/stable/1449306.

[CR35] Medvedev ON, Norden PA, Krägeloh CU, Siegert RJ (2018). Investigating unique contributions of dispositional mindfulness facets to depression, anxiety, and stress in general and student populations. Mindfulness.

[CR36] Medvedev ON, Cervin M, Barcaccia B, Siegert RJ, Roemer A, Krägeloh CU (2021). Network analysis of mindfulness facets, affect, compassion, and distress. Mindfulness.

[CR37] Medvedev ON, Dailianis AT, Hwang YS, Krägeloh CU, Singh NN (2021). Applying generalizability theory to the self-compassion scale to examine state and trait aspects and generalizability of assessment scores. Mindfulness.

[CR38] Moon HG (2019). Mindfulness of death as a tool for mortality salience induction with reference to terror management theory. Religions.

[CR39] Neff KD (2003). Development and validation of a scale to measure self-compassion. Self and Identity.

[CR40] Niemiec CP, Kashdan TB, Breen WE, Brown KW, Cozzolino PJ, Levesque-Bristol C, Ryan RM (2010). Being present in the face of existential threat: The role of trait mindfulness in reducing defensive responses to mortality salience. Journal of Personality and Social Psychology.

[CR41] Podsakoff PM, MacKenzie SB, Podsakoff NP (2012). Sources of method bias in social science research and recommendations on how to control it. Annual Review of Psychology.

[CR42] Pyszczynski T, Greenberg J, Solomon S (1997). Why do we need what we need? A terror management perspective on the roots of human social motivation. Psychological Inquiry.

[CR43] Pyszczynski T, Greenberg J, Solomon S (1999). A dual process model of defense against conscious and unconscious death-related thoughts: An extension of terror management theory. Psychological Review.

[CR44] Pyszczynski T, Greenberg J, Solomon S, Arndt J, Schimel J (2004). Why do people need self-esteem? A theoretical and empirical review. Psychological Bulletin.

[CR45] Pyszczynski T, Lockett M, Greenberg J, Solomon S (2021). Terror management theory and the COVID-19 pandemic. Journal of Humanistic Psychology.

[CR46] Raes F, Pommier E, Neff KD, Van Gucht D (2011). Construction and factorial validation of a short form of the self-compassion scale. Clinical Psychology & Psychotherapy.

[CR47] Schultz DM, Arnau RC (2019). Effects of a brief mindfulness induction on death-related anxiety. OMEGA-Journal of Death and Dying.

[CR48] Sedikides C, Gregg AP (2008). Self-enhancement: Food for thought. Perspectives on Psychological Science.

[CR49] Truong QC, Krägeloh CU, Siegert RJ, Landon J, Medvedev ON (2020). Applying generalizability theory to differentiate between trait and state in the Five Facet Mindfulness Questionnaire (FFMQ). Mindfulness.

[CR50] Vetter T, Oberhammer G (1995). Bei Lebzeiten das Todlose erreichen, Zum Begriff Amata im Alten Buddhismus. Im Tod gewinnt der Mensch sein Selbst, das Phänomen des Todes in asiatischer und abendländischer Religionstradition.

[CR51] Waldschmidt E (1944). Die Überlieferung vom Lebensende des Buddha, eine vergleichende Analyse des Mahāparinirvāṇasūtra und seiner Textentsprechungen, erster Teil.

[CR52] Wasson RS, Barratt C, O’Brien WH (2020). Effects of mindfulness-based interventions on self-compassion in health care professionals: A meta-analysis. Mindfulness.

[CR53] Wong PT, Tomer A (2011). Beyond terror and denial: The positive psychology of death acceptance. Death Studies.

